# The effect of food anticipation on cognitive function: An eye tracking study

**DOI:** 10.1371/journal.pone.0223506

**Published:** 2019-10-14

**Authors:** Michelle S. Segovia, Marco A. Palma, Rodolfo M. Nayga

**Affiliations:** 1 Division of Applied Social Sciences, University of Missouri, Columbia, Missouri, United States of America; 2 Department of Agricultural Economics, Texas A&M University, College Station, Texas, United States of America; 3 Department of Agricultural Economics and Agribusiness, University of Arkansas, Fayetteville, Arkansas, United States of America; University of Florida, UNITED STATES

## Abstract

By randomizing the order in which participants perform a cognitive test and a food choice task in a controlled experiment, we investigate whether cognitive capacity can be enhanced by the simple act of anticipating food intake. Our findings show that overweight and obese participants exhibit an anticipatory food reward effect, which helped enhance their mental resources and improve their performance in a cognitive test. However, we find no anticipation effect among normal weight participants. Furthermore, eye tracking data reveal that food temptation, in the form of visual attention and emotional arousal is higher for overweight and obese individuals when they are cognitively impaired.

## Introduction

Resource scarcity in the form of financial constraints, time pressure, sleep deprivation, and high cognitive load can severely impede cognitive capacity, resulting in suboptimal behavior [1–6]. Scarcity, of any kind, creates a tendency to sequester all available mental resources to focus on the most salient and immediate problem, leaving less available resources for other functions [2]. Scarcity affects decision making in multiple domains [7, 8]. For instance, low-income individuals are often preoccupied about financial and budgetary concerns which makes them more likely to take high-interest loans [2], purchase lottery tickets [9], fail to enroll in welfare assistance programs[10], and make shortsighted economic decisions (i.e. be more impatient) [11, 12]. Restrained eaters focus more on food-related cues [13], are more impatient [14], and overeat under high cognitive load [15]. Likewise, individuals experiencing conditions of ego depletion and extreme time pressure cooperate less in social dilemma games [16], while the sleep deprived make more risky choices in gambling tasks [17–20].

A key finding from previous literature is that cognitive or mental resources can be replenished through a resting period or by supplementation of glucose [21, 22]. This phenomenon has been frequently observed in laboratory settings where subjects are asked to consume a snack to help them restore their blood glucose to normal levels. The results from these studies report an increase in cognitive performance during real effort tasks following glucose supplementation [23, 24]. For example, Massicampo and Baumeister [24] show that participants who are depleted but consume a glucose drink are less prone to rely on heuristic, low-effort decision making, compared to those who drink a placebo beverage. Dickinson et al. show that glucose enriched participants are more likely to make Bayesian choices compared to no-glucose participants [25]. Furthermore, Danziger et al. [26] find that the proportion of favorable judicial rulings fluctuates in relation to the time in which judges take a food break. They attribute this effect to cognitive depletion. However, they are unable to identify whether these fluctuations are due to resource replenishment by eating, resting, or a combination of both.

Much of the aforementioned research suggests that glucose ingestion boosts performance on cognitive and physical tasks [27, 28]. However, this hypothesis has been challenged, both empirically and theoretically [29, 30]. An alternative motivational hypothesis for the effect of glucose on performance suggests that glucose can enhance (physical or cognitive) performance even when it is not ingested [31] by activating brain regions associated with *motivation* and *reward* responses [32, 33]. To evaluate this hypothesis, researchers often instruct participants to rinse their mouths with, but not ingest, glucose solutions prior to performing intense physical or cognitive activities. The results from these studies suggest significant increases in the performance of subjects who rinse their mouths with glucose solutions compared to those who use placebo solutions [34–36]. This effect is attributed to motivational rather than metabolic (i.e. changes in blood glucose levels) processes [31]. In fact, neuroimaging studies have suggested a specific origin for this nonenergetic effect: glucose mouth rinses activate dopaminergic pathways in the *striatrum*—a brain region associated with responses to reward [37] and motivation [32, 33]. The results of these studies suggest that sensing glucose in the mouth (without swallowing), regardless of whether it is consumed or not provides a reward mechanism and boost performance.

Although glucose has been previously linked to metabolic and motivational roles for glucose in performance enhancement, we investigate whether cognitive resources can be replenished by the simple act of *anticipating* food intake, prior to sensing or consuming actual food. This question is important because a necessary condition in the addiction literature relates to the dopamine reward system, which intensifies the “reward” in anticipation of the addictive action and not necessarily in the action itself [38, 39]. In this regard, recent neurobiological evidence has shown that obese individuals are prone to an “anticipatory food reward”, in which they exhibit greater activity in somatosensory and gustatory brain regions in response to anticipating food intake [40–42]. These brain regions are responsible for encoding the sensory and hedonic aspects of food palatability [40], meaning that obese people derive more pleasure from the desire to eat food than from the actual act of eating.

Using a controlled laboratory experiment, we test the causal relationship of an “anticipatory food reward” effect on cognitive function by randomizing the order in which subjects perform a cognitive test and a food choice task. Our main hypothesis is that subjects who perform the food choice task prior to a cognitive test will experience an anticipatory effect since they are aware that they will imminently consume a food snack. If an anticipatory food reward effect exist, then we should observe an increase in the cognitive performance of individuals in the food anticipation treatment. The results show that overweight and obese subjects experience an anticipatory food reward effect, which enhances their cognitive capacity after merely choosing a food snack that would be readily available for them to eat after the experiment. In other words, overweight and obese subjects who complete the food choice task before the cognitive test perform significantly better in a cognitive ability test than those who complete the cognitive test first. This effect is not present in normal weight individuals, who performed identically in the cognitive test regardless of the order of the tasks. The results from our study imply that food anticipation alone may be enough to *replenis*h the cognitive resources of overweight and obese individuals but not for normal weight individuals.

Finally, we use eye tracking to monitor the eye movements of participants while they are exposed to pictures of binary food menus. We use two metrics–total visit duration and pupil dilation–to examine the effects of an anticipatory food reward effect on visual attention (temptation) and emotional arousal of individuals towards the food products. The findings suggest that in the presence of an anticipatory food reward effect, obese individuals do not only exhibit less arousal towards food, but they also shift their attention towards lite snacks.

## Methods

### Participants

The sample consists of 182 students from a large university in the southwestern United States. Students are recruited as subjects since they are relatively homogeneous in terms of demographic and socio-economic characteristics [43], allowing us to make treatment comparisons across BMI categories. Participants are recruited using bulk emails and receive a show-up compensation fee of $20 for participating in the experiment. The experimental sessions are conducted from July to September 2016, one-person at a time in order to collect eye tracking data, during different times of the day (from 8:00 am to 8:00 pm) to control for time-of-the-day effects. Each session lasts around 60 minutes. To qualify for the study, subjects have to be at least 18 years old, must not have a history of psychiatric or eating disorders or known food allergies. The study is approved by the Texas A&M Institutional Review Board (Protocol# 2016-0059D) and it is carried out in accordance with relevant guidelines and ethical standards. In addition, written informed consent is obtained from all participants prior to the sessions. Participants are instructed to refrain from eating for at least three hours prior to their assigned experimental session. The three-hour food deprivation period is selected to mimic the hunger state experienced between meals and to ensure a similar state of hunger across participants and conditions. We check for fasting compliance by asking participants to report the time at which they consume their last meal that day in the demographic/behavioral questionnaire given at the end of the session. Moreover, participants are asked to report their level of hunger on a scale from 1 to 9 (1 = Not at all, and 9 = Extremely hungry) at the beginning of the session, following Ashton (2015). On average, participants report fasting for 6 hours (*SD* = 3.5) prior to the experiment and facing a mild state of hunger at the beginning of the session (*M* = 5.12, *SD* = 2.08).

### Experimental design and procedures

Participants complete a cognitive performance test and a food choice task in randomized order. This generates the two conditions needed to test for the presence of an anticipatory food reward effect on cognitive function. In the first condition, known as the “*anticipatory effect”* condition, subjects complete the food choice task prior to the cognitive test, hence, they have the possibility to experience a food anticipation effect. Subjects in the second (control) condition, known as the “*no anticipatory effect”* condition, perform the cognitive test first, thus, they could not anticipate food consumption while performing the cognitive task.

#### Cognitive performance test

The cognitive performance task incorporates 24 problems from the Raven’s Progressive Matrices test, which is a computer-based test that has been validated as a measure of fluid intelligence (cognitive function) independent of acquired knowledge [44, 45]. The Raven’s problems are displayed individually in order of difficulty. For each test item, subjects are asked to analyze a geometric pattern and identify the missing element that completes the pattern of shapes (see S1 File for an example). After going through an example, participants have 16 minutes to answer the 24 questions.

#### Food choice task

The food choice task presents subjects with 20 binary choice sets. Each choice trial starts with a fixation point slide (2 seconds), followed by a stimulus (two food product images, 8 seconds), a choice decision (no time restriction), and an inter-stimulus slide (0.75 seconds). In each choice trial, subjects are presented with an image of a snack and the “lite” version of the same snack which contains less calories (e.g. original vs. light Yoplait vanilla yogurt, see S1 File for an example, and S1 Table for the list of products) and are asked to choose which of the two snacks they would prefer to eat. All snacks are ready-to-eat items that are carefully selected to be similar, except for the number of calories (they are similar in terms of price, brand, packaging, and flavor). However, the food choices give participants a menu of options to choose from in order to appeal to a variety of food snacks. The position of the food images on the screen is randomized across choice sets; that is, half the choice sets display the lite snack on the right hand side and the regular snack on the left hand side of the screen, the other half display the snacks in the opposite position. We incentivize the food choice task by randomly choosing one of the 20 choice sets as binding. The binding decision is randomly determined by the participant at the end of the experiment using a bingo cage that contains 20 balls numbered 1–20. Each participant withdraws a ball from the bingo cage to determine the binding choice trial and has to eat the snack they chose in the binding decision before leaving the lab.

#### Post-experiment tasks

After completing the two tasks, participants fill out a demographic and behavioral survey. Then, their actual weight and height are collected by two experimenters using a calibrated digital scale and a measuring tape, respectively. Finally, subjects eat the snack they chose in the binding choice set, after which they receive their payment and are escorted out of the laboratory.

#### Eye tracking measures

During the food choice task, eye movements are recorded with a Tobii TX300 desk-mounted eye tracker (Tobii 2014) at a sampling rate of 120 Hz (eye movements are also recorded during the cognitive test but this data is not included in this paper). A nine-point calibration procedure is performed before each recording to ensure the accuracy of the eye tracking metrics [46]. The eye tracker monitor is placed against a beige background and no other stimuli are present that could distract the participant’s attention. The participant’s position is adjusted and the lab illumination is kept constant for good gaze tracking. The visual stimuli are presented on a 23 inch monitor with a screen resolution of 1920 x 1080 pixel and a reaction time of 5 ms. iMotions software platform is used to displayed the stimuli on the screen (iMotions 2014).

Two eye tracking metrics, total visit duration and pupil dilation, are used to assess objective visual attention (temptation) and emotional arousal experienced by participants while performing the food choice task. To analyze total visit duration (TVD), we define two static Areas of Interest (AOIs) for each stimulus, one AOI covering the regular snack and another covering the lite snack image. All AOIs are consistent in terms of size (width and height) and shape (rectangular). The AOIs are used to determine the amount of time (based on fixations) subjects spend on each food image. From the total visit duration time to the presented stimulus, the proportion of gazing to each AOI (food image) is computed. Using the total visit duration time spend in each AOI, we calculate the difference in the time spent between the *lite* and *regular* snacks, which we refer to as δTVD. That is, a positive value indicates higher visual duration toward the lite snacks, while a negative value implies higher visual duration toward the regular snacks. To measure pupil dilation, we calculate the average pupil size (in millimeters) between subjects’ left and right eye while looking at each food stimulus (20 food images). Larger pupil size is indicative of higher emotional arousal toward the snacks.

#### Analysis

We perform our analysis of cognitive performance and eye tracking using non-parametric Wilcoxon Rank-Sum tests (results hold when using parametric tests, i.e. *t*-tests, and are available in S2 Table). We then strengthen the results from the descriptive analysis using panel logit models and ordinary least square (OLS) regressions, in which we control for individual heterogeneity (i.e. standard errors clustered at the individual level).

## Results and discussion

Our study includes 182 participants, with 105 in the *no anticipatory effect* condition and 77 participants in the *anticipatory effect* condition. Nearly half of the participants are male (49.7%), with an average age of 23.4 years and an average family income of $40,275. On average, 34.6% identify themselves as White, 37.4% as Asians, 19.2% as Hispanic, and 8.8% as non-White, non-Asians, and non-Hispanic. Participant’s body mass index (BMI) is calculated by collecting their height and weight and using the standard formula: *kg/m*^*2*^, to classify participants as either normal weight (≤ 24.9), overweight (25–29.9), or obese (≥ 30), following the National Institutes of Health guidelines [47]. The average BMI of the sample is 24.7, which is borderline normal-overweight, with 116 participants in the normal weight category, 41 overweight, and 25 obese. Due to random assignment of the treatments, the distribution of weight status is balanced across treatments, with a larger proportion of normal weight individuals in both treatments (S3 Table). Moreover, there is no difference in the proportion of male and White individuals between treatments (*p ≥* 0.10 for all Wilcoxon rank-sum tests). Self-reported hunger levels are collected in the demographic/behavioral survey using a 9-point rating scale. Using Wilcoxon rank-sum tests, we find no statistical differences (*p ≥* 0.10 for all tests) in the reported level of hunger across weight categories and across treatment conditions, meaning that all subjects experienced a similar state of hunger upon arrival to the lab. S3 Table provides the descriptive statistics for several demographic and behavioral measures obtained from the questionnaire, as well as a balance check across conditions.

Our main hypothesis is that subjects who perform the food choice task prior to the cognitive test will experience an anticipatory food reward effect, which if present, will enhance their cognitive ability. If this is the case, we expect a significant increase in the cognitive performance (as measured by the Raven’s test) of subjects in anticipation of food consumption.

**Result 1**: *An anticipatory food reward effect improves the cognitive performance of overweight and obese individuals*.

**Fig 1:**
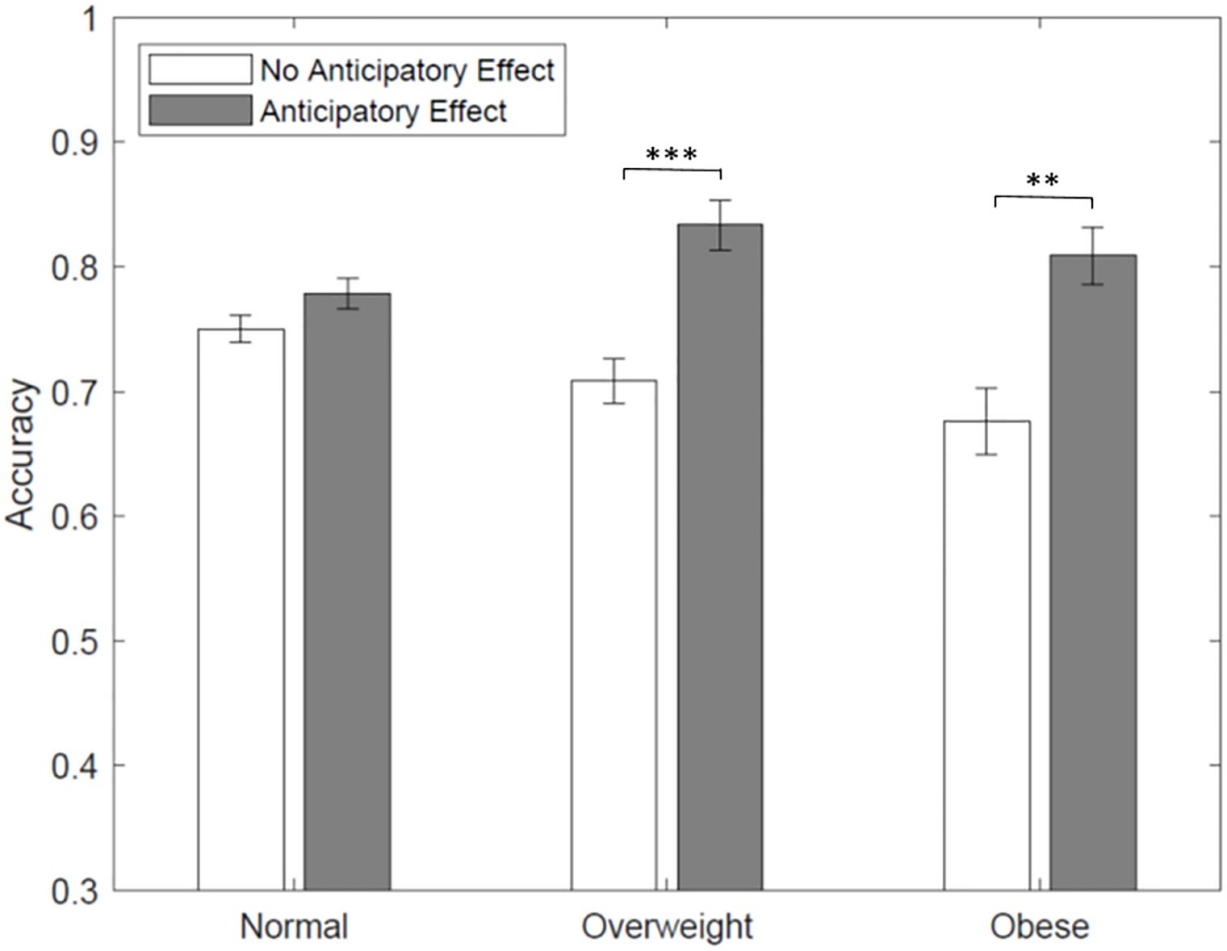
Accuracy on Raven’s test by treatment and BMI category. Accuracy represents the proportion of correct answers in the Raven’s (cognitive) test; *** *p* < 0.01, ** *p* < 0.05, and * *p* < 0.10; mean comparisons are performed using Wilcoxon rank-sum tests.

The cognitive performance in the Raven’s test by BMI is presented in Fig 1. Accuracy represents the proportion of correct answers in the Raven’s test. The results show that the performance of normal weight individuals in the cognitive test does not change by the order in which the cognitive test and food choice task is presented (*Z* = 1.354, *p* = 0.176, Wilcoxon rank-sum test). This means that normal weight individuals do not exhibit a food reward anticipation effect. However, overweight and obese subjects who complete the food choice task prior to the cognitive test perform significantly better than those who complete the tasks in the opposite order (*Z* = 2.799, *p* = 0.005 for overweight, and *Z* = 2.312, *p* = 0.021 for obese, Wilcoxon rank-sum tests). This result provides a clear indication of the presence of an anticipatory food reward effect for overweight and obese individuals. Recall that overweight and obese individuals are informed that they would be consuming a snack at the end of the experiment. These results show that the expectation of the imminent snack consumption help enhance their mental resources and performance in the test. A potential explanation for this effect relates to the motivational role of glucose on performance. It is possible that food anticipation might have signaled the possibility of reward (i.e. the immediate availability of food) which boosts the mental activity of overweight and obese participants. Interestingly, this effect is only found among overweight and obese participants. The results of our controlled experiment are supported by previous neurobiological evidence suggesting that obese individuals anticipate reward from food intake compared to lean individuals [40].

Although the descriptive analysis presented so far carries strong results, it fails to account for individual heterogeneity and interaction effects between conditions. Several Panel Logit regression specifications on cognitive ability are estimated and the results are presented in Table 1. The dependent variable is a dummy variable that takes the value of 1 for correct answers in the cognitive test. The independent variables (omitted) include anticipatory effect (no anticipatory effect), overweight and obese (normal weight), and interaction terms. As shown in Table 1, the specifications in the first two columns include the treatment (presence or absence of anticipatory food reward) and weight status as the only explanatory variables. The specification in column 3 includes interactions between weight status and the treatments. The regression estimation results support our main findings of an anticipatory food reward effect among overweight and obese individuals. This effect is captured by positive and significant coefficients on the interaction between weight status (overweight and obese) and the anticipatory effect. We conclude that in the presence of an anticipatory food reward effect, the cognitive capacity of overweight and obese individuals improves their cognitive performance in the Raven’s test.

**Table 1:** The impact of food anticipation on cognitive ability: Panel logit.

Variable	Coefficient (Std. Error)		Coefficient (Std. Error)		Coefficient (Std. Error)	
	(1)		(2)		(3)	
ANTICIPATORY EFFECT	0.361	***	0.364	***	0.158	
	(0.074)		(0.110)		(0.135)	
Overweight			-0.010		-0.224	
			(0.135)		(0.166)	
Obese			-0.137		-0.384	*
			(0.155)		(0.227)	
Overweight x Anticipatory Effect					0.596	**
					(0.277)	
Obese x Anticipatory Effect					0.567	**
					(0.294)	
Constant	1.064	***	1.083	***	1.167	***
	(0.074)		(0.085)		(0.094)	
Observations	4,368		4,368		4,368	
Log-Likelihood	-2372.34		-2371.96		-2368.75	

*Notes*: Standard errors in parentheses are clustered at the individual level;

*** *p* < 0.01,

** *p* < 0.05,

* *p* < 0.10;

number of observations equals number of subjects × 24 Raven’s problems.

In order to investigate the potential mechanism behind the results, we use two eye tracking metrics–namely pupil dilation and δTVD—to examine the effects of anticipatory food reward on visual attention (temptation) and emotional arousal towards the food snacks. These metrics are obtained by recording subjects’ eye movements while they perform the food choice task.

**Result 2**: *An anticipatory food reward effect shifts the visual attention of overweight and obese individuals towards lite food*.

**Fig 2:**
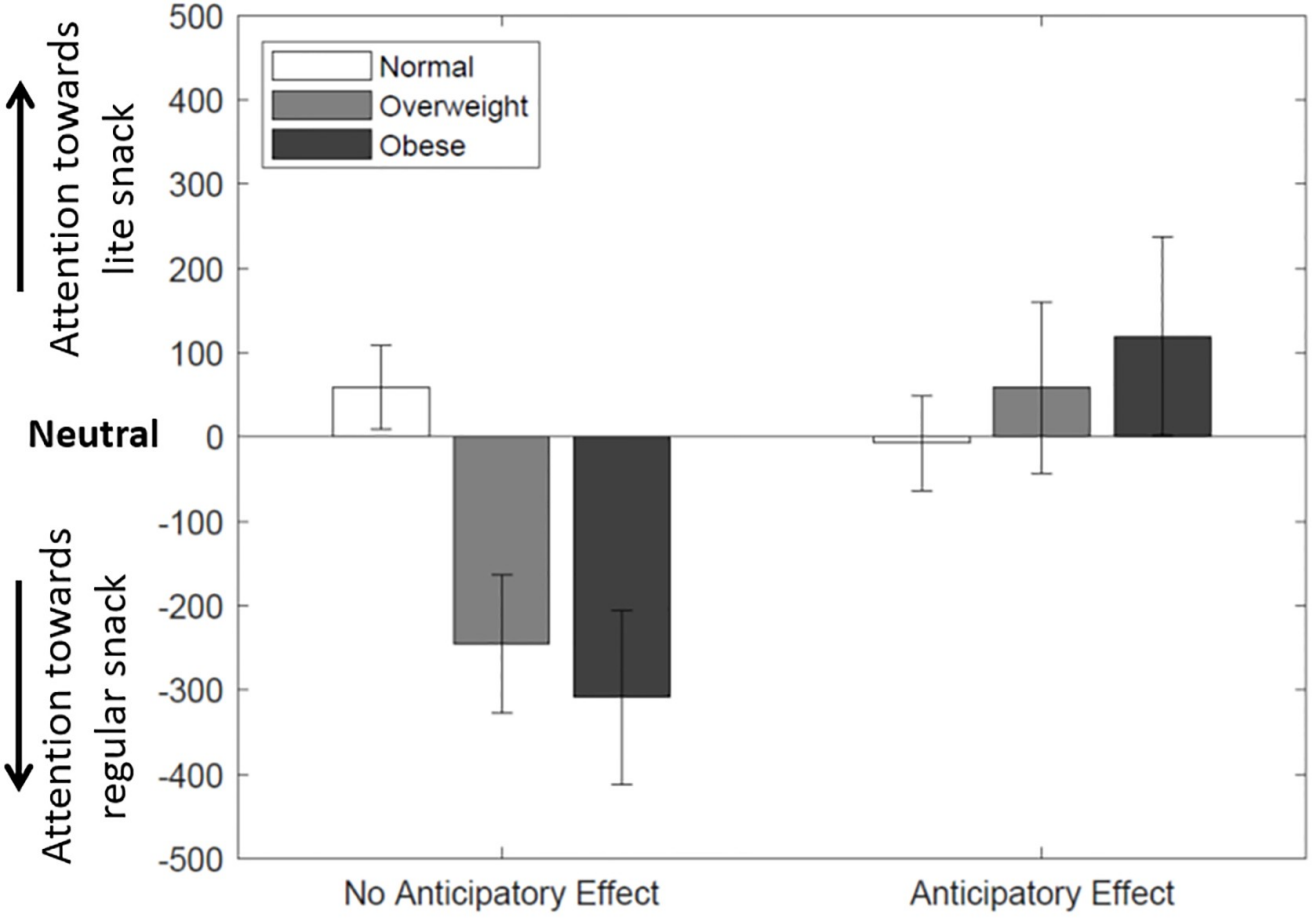
Attention level (temptation) towards food products by treatment and BMI category. Attention (temptation) is measured as the difference in time visit duration between the regular *versus* the lite snacks (δTVD); positive values means that subjects spend more time looking at the lite snacks, while negative values implies the opposite.

Fig 2 plots the difference in total visit duration between lite and regular snacks, δTVD, by treatment and weight status. Recall that a negative value indicates that subjects spend more time looking at the regular snacks, while a positive value implies the opposite. This metric has been previously used as an index of maintained attention on food-related stimuli [48, 49]. The results presented in Fig 2 show that overweight and obese subjects in the *no anticipatory effect* condition spend more time looking at the regular snacks compared to normal weight individuals (*Z* = 2.072, *p* = 0.038 for overweight, and *Z* = 2.446, *p* = 0.014 for obese, Wilcoxon rank-sum tests). This result supports the findings of Nijs et al. [50] suggesting a higher level of automatic attention to food related stimuli in food deprived overweight and obese subjects compared to normal weight subjects. While overweight and obese subjects exhibit more temptation towards regular snacks in the *no anticipatory effect* condition, their attention shifts towards the lite snacks after experiencing an anticipatory reward effect (*Z* = 1.380, *p* = 0.168 for overweight, and *Z* = 1.849, *p* = 0.063 for obese, Wilcoxon rank-sum tests).

The results from Fig 2 are reinforced by the OLS regressions reported in Table 2. Here, the dependent variable is the difference in total visit duration between the lite and regular snacks (δTVD), with a positive value indicating higher attention toward the lite snack. Specification (1) includes the treatment dummy, Anticipatory Effect, which is not significant. A positive albeit insignificant effect persists after controlling for weight status (specification 2). However, when accounting for heterogeneous treatment effects (specification 3), we find negative and significant coefficients for Overweight and Obese, meaning that they exhibit more temptation toward regular snacks compared to normal weight subjects. We also find positive and significant coefficients for the interactions, Overweight × Anticipatory and Obese × Anticipatory Effect. These indicate that overweight and obese individuals who experience an anticipatory food reward effect spend more time looking at the lite snacks than normal weight subjects do.

**Table 2:** The impact of food anticipation on attention to food: OLS.

Variable	Coefficient (Std. Error)	Coefficient (Std. Error)	Coefficient (Std. Error)	
	(1)	(2)	(3)	
ANTICIPATORY EFFECT	87.097	83.094	-66.535	
	(82.329)	(81.646)	(97.367)	
Overweight		-159.315	-304.673	**
		(106.641)	(138.939)	
Obese		-137.696	-367.903	**
		(119.798)	(149.093)	
Overweight x Anticipatory Effect			370.654	*
			(210.443)	
Obese x Anticipatory Effect			494.863	**
			(231.018)	
Constant	-61.709	-5.211	59.284	
	(52.022)	(53.088)	(54.449)	
Observations	3,640	3,640	3,640	
R-squared	0.000	0.001	0.003	

*Notes*: Standard errors in parentheses are clustered at the individual level;

*** *p* < 0.01,

** *p* < 0.05,

* *p* < 0.10;

number of observations equals number of subjects × 20 food choices.

The results on visual attention are further supported by a second eye tracking metric, measured as changes in the subjects’ pupil size. Pupil dilation has been associated with emotional reactions and information processing [51, 52]. In the context of decision-making, pupillary activity has been found to be a reliable correlate of emotional engagement or arousal [53, 54], as the pupil dilates more when subjects exhibit higher approach towards the stimuli, regardless of their hedonic valence [55]. For example, an increase in pupil size has been found in both adults and infants as response to positive and negative stimuli [56], unusual and novel stimuli [57, 58], and threatening stimuli [59]. In the present study, average pupil size or changes in pupillary dilation, are used as indicative of emotional arousal exhibited by participants while looking at the snacks in the food choice task. Importantly, lighting conditions and computer background colors are identical across the control and treatment, since the only change in the anticipation treatment is the order in which it is performed.

**Result 3**: *An anticipatory food reward effect reduces emotional arousal towards food among obese individuals*.

**Fig 3:**
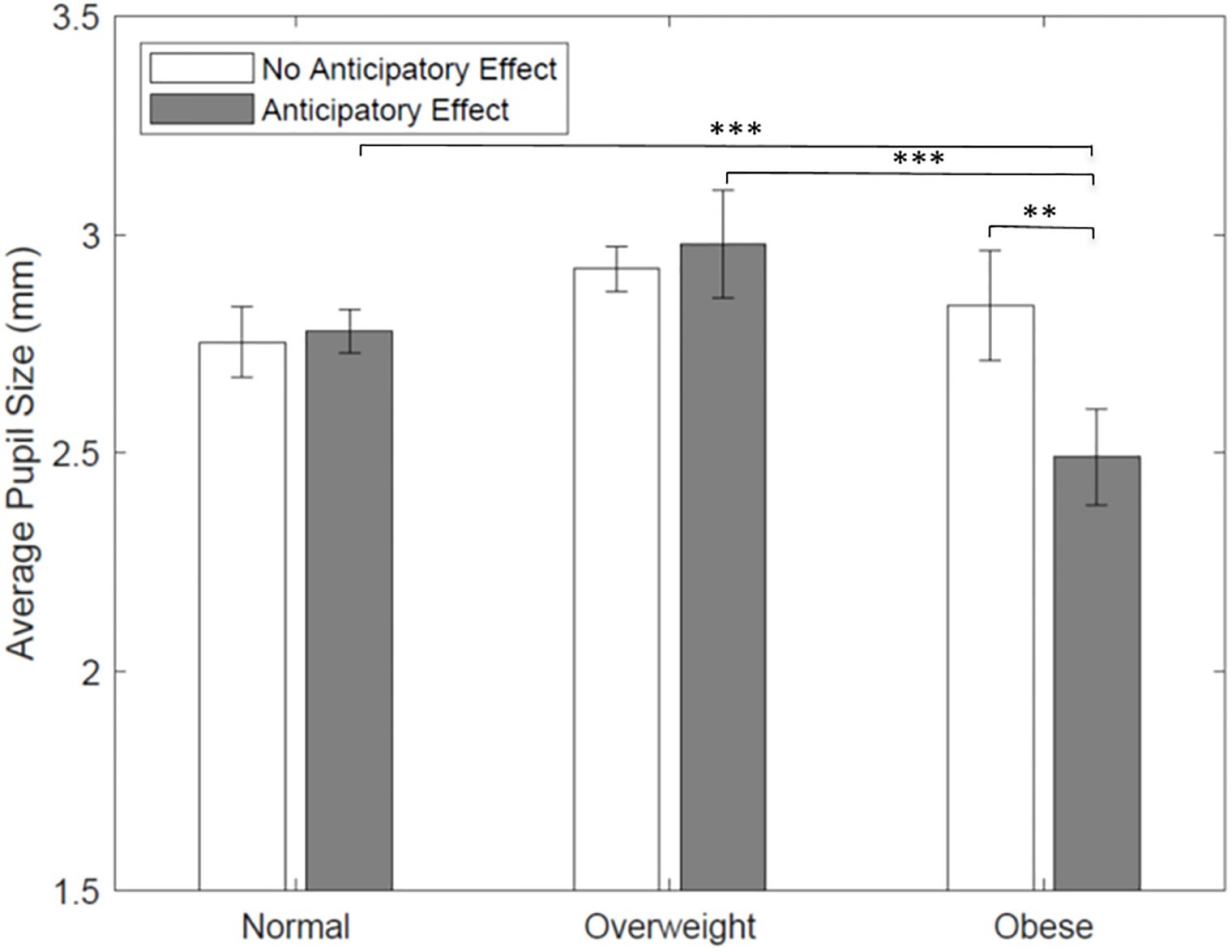
Average pupil size during food choice task by treatment and BMI category. Average pupil size is used as indicative of emotional arousal exhibit by subjects while looking at the snacks in the food choice task; *** *p* < 0.01, ** *p* < 0.05, and * *p* < 0.10; mean comparisons are performed using Wilcoxon Rank-Sum tests.

The results for the average pupil size are shown by treatment and BMI category in Fig 3. For normal weight and overweight individuals, no significant effects are found in their pupil size which measures their emotional arousal across treatments (*p* > 0.10 in all Wilcoxon rank-sum tests). However, there are significant differences among obese individuals. Specifically, the average pupil size is significantly lower for obese subjects who experience an anticipatory food reward effect (Z = -2.339, *p* = 0.019, Wilcoxon rank-sum test), meaning that when the obese anticipate food intake, they exhibit lower arousal towards the snacks. The OLS regressions in Table 3 formally present the heterogeneous treatment effects of food anticipation on average pupil size of overweight and obese subjects (specification 3). The differences by weight status, observed in the previous analysis, are evident here from the fact that the coefficient on the interaction between Obese and Anticipatory Effect is negative and significant. This strengthens our result that, in the presence of food anticipation, obese individuals exhibit less emotional arousal toward food compared to normal weight individuals.

**Table 3:** The impact of food anticipation on average pupil size during food task: OLS.

Variable	Coefficient (Std. Error)		Coefficient (Std. Error)		Coefficient (Std. Error)	
	(1)		(2)		(3)	
ANTICIPATORY EFFECT	-0.071		-0.061		-0.027	
	(0.061)		(0.061)		(0.076)	
Overweight			0.149	**	0.125	
			(0.068)		(0.076)	
Obese			-0.105		0.051	
			(0.093)		(0.128)	
Overweight x Anticipatory Effect					0.072	
					(0.149)	
Obese x Anticipatory Effect					-0.341	**
					(0.173)	
Constant	2.846	***	2.823	***	2.809	***
	(0.041)		(0.051)		(0.057)	
Observations	3,617		3,617		3,617	
R-squared	0.005		0.030		0.046	

*Notes*: Standard errors in parentheses are clustered at the individual level;

*** *p* < 0.01,

** *p* < 0.05,

* *p* < 0.10;

number of observations equals number of subjects × 20 food choices.

Overall, the observed pattern of attention allocation in this study indicates that in the absence of an anticipatory food reward effect, obese individuals do not only exhibit more arousal towards food, but they also pay more attention and are more tempted by regular (higher caloric) food products. These effects are attributed to the cognitive impairment subjects experienced in the absence of food anticipation.

## Conclusion

The current research contributes to advancing the understanding of the effects of food anticipation on cognitive ability. The results of the experiment show that overweight and obese individuals exhibit an anticipatory food reward effect, which helped enhance their mental resources and improve their performance in a cognitive test. These behavioral findings are supported by eye tracking data, which reveal that temptation–in the form of visual attention and emotional arousal–is higher under low cognitive resources.

This study provides two important contributions. First, our findings show that an anticipatory food reward effect can help offset the cognitive cost associated with hunger by enhancing the mental resources of overweight and obese individuals. Although alternative methods have proven useful in restoring mental resources–such as glucose rinsing and supplementation, rest, and positive mood [24–26]–we find that the simple act of anticipating food consumption is also effective in this regard. This result offers behavioral support to neuroscience evidence suggesting the presence of an anticipatory food reward effect among obese individuals [38, 40]. Second, this is the first study, to our knowledge, that uses eye tracking to examine the relationship between visual attention, cognitive function, and food-related decision making. The findings show that overweight and obese individuals seem to be particularly responsive to food stimuli when cognitively impaired. While overweight and obese subjects draw more attention towards unhealthy (regular) snacks, higher emotional arousal towards the food is exhibited by the obese only.

The current research offers implications for policy makers designing programs to promote the consumption of healthy foods. For example, in contexts where the anticipation of food reward is present, commitment devices can be incorporated in an effort to limit the availability of food options to healthy foods. The results from this experiment show that even anticipating the consumption of snacks with relatively low calories, can help restore the mental resources of overweight and obese individuals. Since an anticipatory food reward effect can only be achieved by imminently eating, committing to healthy snacks in advance (i.e. knowing exactly what to eat) might offer potential benefits [60]. For example, companies that offer (tax-deductible) free snacks to their employees could potentially restrict the type of food items available to employees to be healthier. This could be seen as a strategy to improve workers’ cognitive ability through food anticipation while keeping them healthy. Other commitment devices may include getting rid of sugar and refined foods at home or creating grocery shopping lists to commit to healthy food in advance. In this regard, researchers have found that preordering food or signing up for food box subscriptions may lead to healthier food choices [61, 62]. Whether this type of pre-commitment scheme has potential to increase concentration and boost work productivity is left for future research.

Finally, additional research can evaluate the results using larger sample sizes, particularly for obese participants. Although we employ multiple recruiting methods, there are persistent difficulties in recruiting overweight and obese participants. A potential suggestion for future related work might be to use experimenters with high BMI for the recruitment process (see Brown et al. for a review on effective recruiting strategies for minority groups [63]). On another note, while the cognitive test used in this study did not carry an economic reward, it might be worthwhile to measure cognitive ability using an incentivized task to determine whether the effect of food anticipation on cognitive ability holds under the presence of real rewards or incentives.

## Supporting information

S1 File Experimental instructions. (PDF)

S1 Table List of products used in food choice task. (XLSX)

S2 Table Mean comparisons between non-anticipatory and anticipatory conditions by BMI. (XLSX)

S3 Table Descriptive statistics and balance test across treatment groups. (XLSX)

S1 Data (XLS)

S2 Data (XLS)
