# Supplementary material for: The effect of food anticipation on cognitive function: An eye tracking study
Source: PLoS One. 2019 Oct 14;14(10):e0223506. doi: 10.1371/journal.pone.0223506 (PMC6791586; doi:10.1371/journal.pone.0223506)
Supplement: S1 File — (PDF) [file pone.0223506.s001.pdf]

S1 File. Experimental Instructions

Notes: The order in which the food choice task and cognitive test are performed is randomized across subjects depending on the condition.

[*Screen 1*]

WELCOME!

Thank you for participating in our study. The session will proceed in several stages.

Stage 1: Food Choice Task

Stage 2: Cognitive Test

Stage 3: Receive Product and Payment

Press <Enter> to continue…

[*Screen 2*]

Food Choice Task

This stage will proceed as follows:

1. This stage consists of 20 choice situations.
2. In each trial, you will be presented with two food products.
3. You need to choose which of the products you would prefer to eat.

Press <Enter> to continue…

[*Screen 3*]

4. Your decisions are real. At the conclusion of the experiment, one decision will be randomly selected to be binding.
5. You will receive one single unit of the food product you chose and will have to eat it at the end of today's session.

Press <Enter> to continue…

[*Screen 4-7*]

Example of one of the 20 trials subjects made in the food choice task.

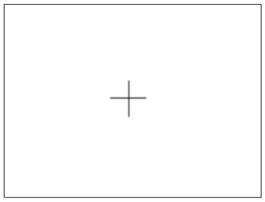

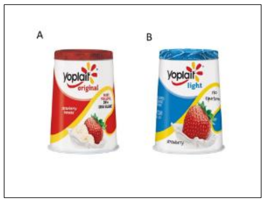

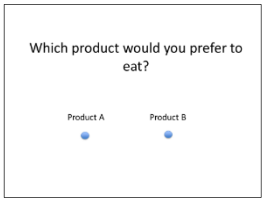

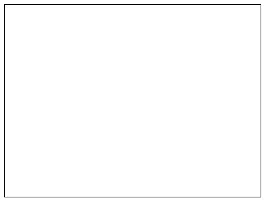

[*Screen 8*]

This concludes this stage of the experiment.

Now we will proceed to the Cognitive Test.

Press <Enter> to continue…

[*Screen 9*]

Cognitive test

This stage will proceed as follows:

1. You will be asked 24 questions.

2. In each question, you will be asked to analyze a geometric pattern and identify the missing part to complete the series.
3. The test takes approximately 16 minutes.

Press <Enter> to continue…

[*Screen 10]*

4. A sample question is shown below, the aim is to identify the most appropriate match out of the given eight options. Here the fourth figure is the most appropriate match among the given options.

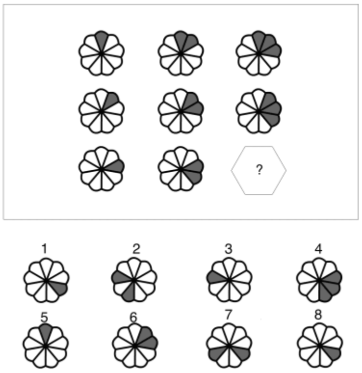

Press <Enter> to continue…

[*Screen 11*]

Example of one of the 24 problems subjects completed in the cognitive test.

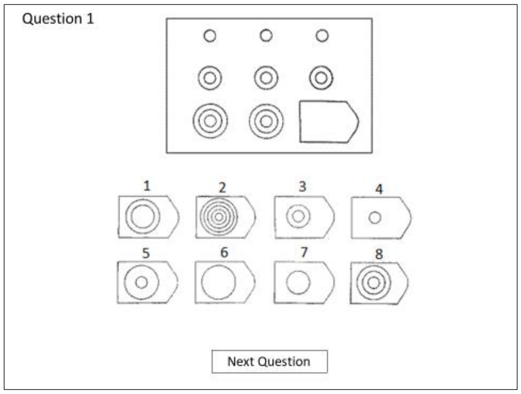

[*Screen 12*]

This concludes today's experiment!

You will now draw the decisions in the food choice task that will be realized.

You will have to consume the chosen food product before leaving the lab.

Finally, you will receive your payment.

Thank you very much for your participation!

<u>Demographic/Behavioral Survey</u>

Please answer the following survey questions.

How often do you exercise? (Include only periods of exercise longer than 20 minutes).

a) Never
b) Once a month
c) Once a week
d) 2-3 times per week
e) 4-6 times per week
f) Once a day
g) More than once a day

Do you currently smoke cigarettes?

a) Yes
b) No

Do you consume alcohol?

a) Yes
b) No

How many hours did you sleep last night?

How many days per week do you eat breakfast?

a) 0 days
b) 1 day
c) 2 days
d) 3 days
e) 4 days
f) 5 days
g) 6 days
h) 7 days

At what time did you consume your last meal today?

Rate on the scale from 1 to 9, how hungry were you feeling at the beginning of the session (1= Not at all; 9= Extremely hungry).

Do you currently have a serious health issue?

Please indicate your age in years.

Please indicate your Major by Department.

Please indicate your current academic year.

a) Freshman
b) Sophomore
c) Junior
d) Senior

Please indicate your gender.

a) Male
b) Female

Please indicate your race.

a) Asian/Pacific Islander
b) African American
c) Caucasian/White
d) Native America/Indigenous
e) Hispanic
f) Other (Please list below)

Please indicate your household yearly income for 2015. (Include all forms of income, including salary, interest and dividend payments, tips, scholarship support, student loans, parental support, and allowance)

a) Less than $30,000
b) $30,000 - $39,999
c) $40,000 - $49,999
d) $50,000 - $59,999
e) $60,000 - $69,999
f) $70,000 - $79,999
g) $80,000 - $89,999
h) $90,000 - $99,999
i) $100,000 - $149,999
j) $150,000 or more
